# *In Vivo* Screening Using Transgenic Zebrafish Embryos Reveals New Effects of HDAC Inhibitors Trichostatin A and Valproic Acid on Organogenesis

**DOI:** 10.1371/journal.pone.0149497

**Published:** 2016-02-22

**Authors:** Ling Li, François Bonneton, Marie Tohme, Laure Bernard, Xiao Yong Chen, Vincent Laudet

**Affiliations:** 1 Institut de Génomique Fonctionnelle de Lyon, Université de Lyon, Université Lyon 1, CNRS, INRA, Ecole Normale Supérieure de Lyon, Lyon, France; 2 School of Ecological and Environmental Science, East China Normal University, Shanghai, China; University of Louisville School of Medicine, UNITED STATES

## Abstract

The effects of endocrine disrupting chemicals (EDCs) on reproduction are well known, whereas their developmental effects are much less characterized. However, exposure to endocrine disruptors during organogenesis may lead to deleterious and permanent problems later in life. Zebrafish (*Danio rerio*) transgenic lines expressing the green fluorescent protein (GFP) in specific organs and tissues are powerful tools to uncover developmental defects elicited by EDCs. Here, we used seven transgenic lines to visualize *in vivo* whether a series of EDCs and other pharmaceutical compounds can alter organogenesis in zebrafish. We used transgenic lines expressing GFP in pancreas, liver, blood vessels, inner ear, nervous system, pharyngeal tooth and pectoral fins. This screen revealed that four of the tested chemicals have detectable effects on different organs, which shows that the range of effects elicited by EDCs is wider than anticipated. The endocrine disruptor tetrabromobisphenol-A (TBBPA), as well as the three drugs diclofenac, trichostatin A (TSA) and valproic acid (VPA) induced abnormalities in the embryonic vascular system of zebrafish. Moreover, TSA and VPA induced specific alterations during the development of pancreas, an observation that was confirmed by *in situ* hybridization with specific markers. Developmental delays were also induced by TSA and VPA in the liver and in pharyngeal teeth, resulting in smaller organ size. Our results show that EDCs can induce a large range of developmental alterations during embryogenesis of zebrafish and establish GFP transgenic lines as powerful tools to screen for EDCs effects *in vivo*.

## Introduction

Since the sixties, there is a growing awareness about the increased presence of chemical pollutants in our environment and by their potential impact on humans and wildlife [1]. Twenty years ago, the natural or synthetic substances that could alter the normal function of the endocrine systems of an organism or its descendants were called endocrine disruptors (EDCs) [2,3]. Today, there is a huge research effort to better detect the presence of EDCs in our environment as well as to understand the consequences of exposure to these compounds. The resulting data attract the attention of health authorities and guide their decisions on the use of these chemicals [4,5]. The sources of EDCs are diverse, including pesticides (herbicides, insecticides), plasticizers (phthalates, bisphenol A), pharmaceuticals (contraceptives drugs, antibiotics), consumer products (detergents, cosmetics) or even chemicals from cooking and burning (polycyclic aromatic hydrocarbons), heavy metals (cadmium, copper) and natural plant metabolites (genistein present in soybean). Endocrine disruptors may act directly as agonists or antagonists that interfere with hormone receptor on target cells. They can also act indirectly by altering hormone dynamics in blood circulation, inducing changes in hormone metabolism, or interfering with hormone action [6,7].

It has been demonstrated over the years that EDCs can produce a wide range of reproductive defects, such as infertility, sex reversal, sexual behavior problems and sex organs abnormalities [6]. In human, the Testicular Dysgenesis Syndrome (TDS), which cluster poor semen quality as well as increased testicular cancer and sexual organs abnormalities (hypospadias and cryptorchidism), has been associated with exposure to EDC such as BPA, PCBs, phthalates or dioxins [8]. In several animal models, EDCs exposure have been shown to induce diverse and specific effects on the testis, the ovary and many other reproductive organs and often results in either feminization or masculinization of the exposed animals [9,10]. In accordance with these effects many EDCs have been shown to target the sex steroid receptors, such as the Estrogen Receptors (ERs) and Androgen Receptor (AR) that are known to be critical for sexual differentiation [11–13]. Nevertheless, it is now clear that these reproductive effects are only “the tip of the iceberg” and that EDCs can elicit other effects [6]. In particular, there are increasing evidence that some of these molecules can affect metabolism and may be linked to the increased incidence of obesity and diabetes [4]. Effects on the immune system as well as on behavior are also suspected [14]. It is therefore urgent to study how EDCs can affect biological processes other than reproduction.

In this respect, the developmental effects of EDCs are particularly important to study. Compared to the adult, the embryo does not have a fully functional immune system and metabolism. Also, because organs change rapidly during development through a series of commitments, they are more sensitive to EDCs exposures. For example, BPA exposure results in dose-dependent malformations of the otic vesicle in zebrafish and in *Xenopus* embryos [15]. Furthermore, exposures of embryos to EDCs can have different outcomes than the exposure of adults. First, the adverse effects may occur at lower concentrations than those considered in adult. Secondly, exposure during the early developmental stages may not lead to harmful consequences immediately, but can lead to serious health problems later on in life [16]. These observations define what is called a “developmental window of susceptibility”, which is an important feature of EDCs. In zebrafish, for example, BPA treatments started prior 22 hours post-fertilization (hpf) lead to 85–100% of otolith defects, while treatment started after 22 hpf do not affect embryos. Therefore, BPA affects otolith development in a restricted time window [15]. However, despite the importance of embryonic sensitivity to EDCs, this issue has been relatively understudied, until recently [17].

Zebrafish (*Danio rerio*) has many advantages as a screening model to study the effects of EDCs on vertebrate organogenesis. The embryos are small, transparent and develop rapidly, which is particularly important for an efficient screening [18,19]. In zebrafish, the principal body plan is established within 24 hpf, and most organs are fully developed within 96 hpf [20,21]. Another advantage of zebrafish is the availability of transgenic lines expressing the green fluorescent protein (GFP) under the control of a tissue-specific promoter [22]. Using this system it is possible to have access to the development of a specific organ (e. g. the liver), of a complex structure (e.g. the vasculature) or even of a specific cell type (e.g. the β-cells of the pancreas). With such lines, the effect of a compound on the GFP expression can be visualized in the transparent developing embryo [20].

The aim of our study was to describe the overall developmental defects generated by diverse compounds on the development of specific organs using zebrafish as a model system. In this screen, we used nine molecules that represent different chemical classes, both in terms of use and mode of action. We selected six clear endocrine disruptors used as flame- retardants, herbicides, insecticides or plasticizer as well as a heavy metal. In addition, we selected three drugs used as antibiotic, anticonvulsants and anti-inflammatory. We have monitored the effects of these compounds on seven transgenic lines chosen to allow visualizing different organs on three distinct systems: the vascular (mesodermal), digestive (endodermal) and nervous (ectodermal) systems. The development of the vascular system (vasculogenesis and angiogenesis) is easily observable during the first 5 days of embryogenesis and known to be sensitive to chemical exposure. We found that four chemicals elicited specific developmental effects: Tetrabromobisphenol A (TBBPA), Diclofenac, Trichostatin A (TSA) and Valproic acid (VPA). While TBBPA is a known EDC [23,24], endocrine disrupting activity of TSA and VPA has not been described. We observed that these effects target endodermal organs (liver, pancreas) as well as the vasculature and teeth development. The validity of the effects detected with GFP was verified using whole mount *in situ* hybridization with specific markers. Our results show that EDCs can also act as embryonic disruptors. Our study also establishes zebrafish transgenic lines as powerful tools to screen for rapid *in vivo* screening small molecules and their effects on development.

## Results

### Chemicals screening with zebrafish transgenic lines

In order to test the effects of various compounds on zebrafish transgenic embryos, we have chosen six known EDCs (TBBPA, atrazine, methoxychlor, CdCl_2_, DEHP, chlordecone). In addition, we tested three pharmaceuticals drugs (diclofenac, TSA and VPA) without known effects on endocrine system. Since these compounds are stable in water, we performed a rapid and simple screen using a static non-renewal test that minimizes embryo manipulation. This also ensures that the exposure dose is not superior to the nominal concentration. Since low-dose effects interest us, we used concentrations well below the lethal dose, such that survival rates of treated embryos after one to five days were comparable to the controls. Because we aimed to describe new effects elicited by these compounds on the development of zebrafish embryo, we have chosen a large range of concentrations from 1 nM to 10 μM. We tested these compounds on seven transgenic lines that represent the vascular (blood vessels), digestive (pancreas, liver, pharyngeal tooth) and nervous systems (inner ear) (Table 1). A total of 500 embryos were observed for each molecule. We do not report the effects that were present in less than 70% of treated embryos. This screen revealed that four of the chemicals have detectable effects, two of which on four different organs (Table 2). The endocrine disruptor TBBPA, as well as the tested medicines (diclofenac, TSA and VPA) induced vascular abnormalities (S1 Fig). In addition, TSA and VPA also affected the development of pancreas and liver. Finally, TSA and VPA inhibited pharyngeal tooth formation. Overall, our results demonstrate the efficiency of such a rapid screening for the detection of significant developmental effects of EDCs *in vivo*. Further analyses were performed to validate this conclusion. To achieve this, we focused on TSA and VPA because these compounds exhibit the most diversified and original effects.

**Table 1:** Transgenic lines used in this study.

Transgenic lines	Expression	Observed stages	Reference
Fli1-EGFP	Blood vessels	24–72 hpf	[25]
Insulin-GFP	Endocrine pancreas	24–72 hpf	[26]
Elastase A -EGFP	Exocrine pancreas	72–120 hpf	[27]
LFABP-EGFP	Liver	72–120 hpf	[28]
Dlx2b-EGFP	Pharyngeal tooth, fins, hindgut	72–120 hpf	[29]
α1-tubulin-EGFP	Nervous system	24–72 hpf	[30]
Shh-GFP	Floor plate, ventral brain, hypothalamus	24–72 hpf	[31]
Brn3c-mGFP	Retina, inner ear, lateral line	56–120 hpf	[32]

**Table 2:** Results of the screening with different transgenic lines. Effect (+) or no effect (-) on the GFP expression pattern. nd: not determined.

Molecules	Use	Fli1-EGFP	Insulin-GFP	LFABP-EGFP	Dlx2b-EGFP	α1-tubulin-EGFP	Shh-GFP	Brn3c-mGFP
Trichostatin A (TSA)	Antifungal antibiotic	**+**	**+**	**+**	**+**	**-**	**-**	**-**
Valproic acid (VPA)	Anticonvulsant drug	**+**	**+**	**+**	**+**	**-**	**-**	**-**
Diclofenac	Anti-inflammatory	**+**	**-**	**-**	**-**	**-**	**-**	**-**
Tetrabromobisphenol A (TBBPA)	Flame retardant	**+**	**-**	*nd*	**-**	**-**	**-**	**-**
Cadmium Chloride (CdCl_2)_	Heavy metal	**-**	**-**	**-**	**-**	**-**	**-**	**-**
Bis(Diethylhexyl) Phthalate (DEHP)	Plasticizer	**-**	**-**	**-**	**-**	**-**	**-**	**-**
Atrazine	Herbicide	**-**	**-**	**-**	**-**	**-**	**-**	**-**
Chlordecone	Insecticide	**-**	**-**	**-**	**-**	**-**	**-**	**-**
Methoxychlor	Insecticide	**-**	**-**	**-**	**-**	**-**	**-**	**-**

### HDAC inhibitors VPA and TSA disrupt vascular development

Both VPA and TSA are histone deacetylases (HDAC) inhibitors used as pharmaceutical drugs, with no clear endocrine disrupting activity described. To understand more precisely the nature of the defects induced by VPA and TSA in the vascular system, we monitored the fluorescence in Fli1-EGFP embryos in which 15 kb of the zebrafish *fli1* promoter drive the expression of enhanced green fluorescent protein (EGFP) in all blood vessels throughout embryogenesis [25]. Fli1-EGFP embryos were treated with VPA and TSA from 1 nM to 10 μM at 5 hpf and the fluorescence was analyzed from 24 to 72 hpf.

**Fig 1:**
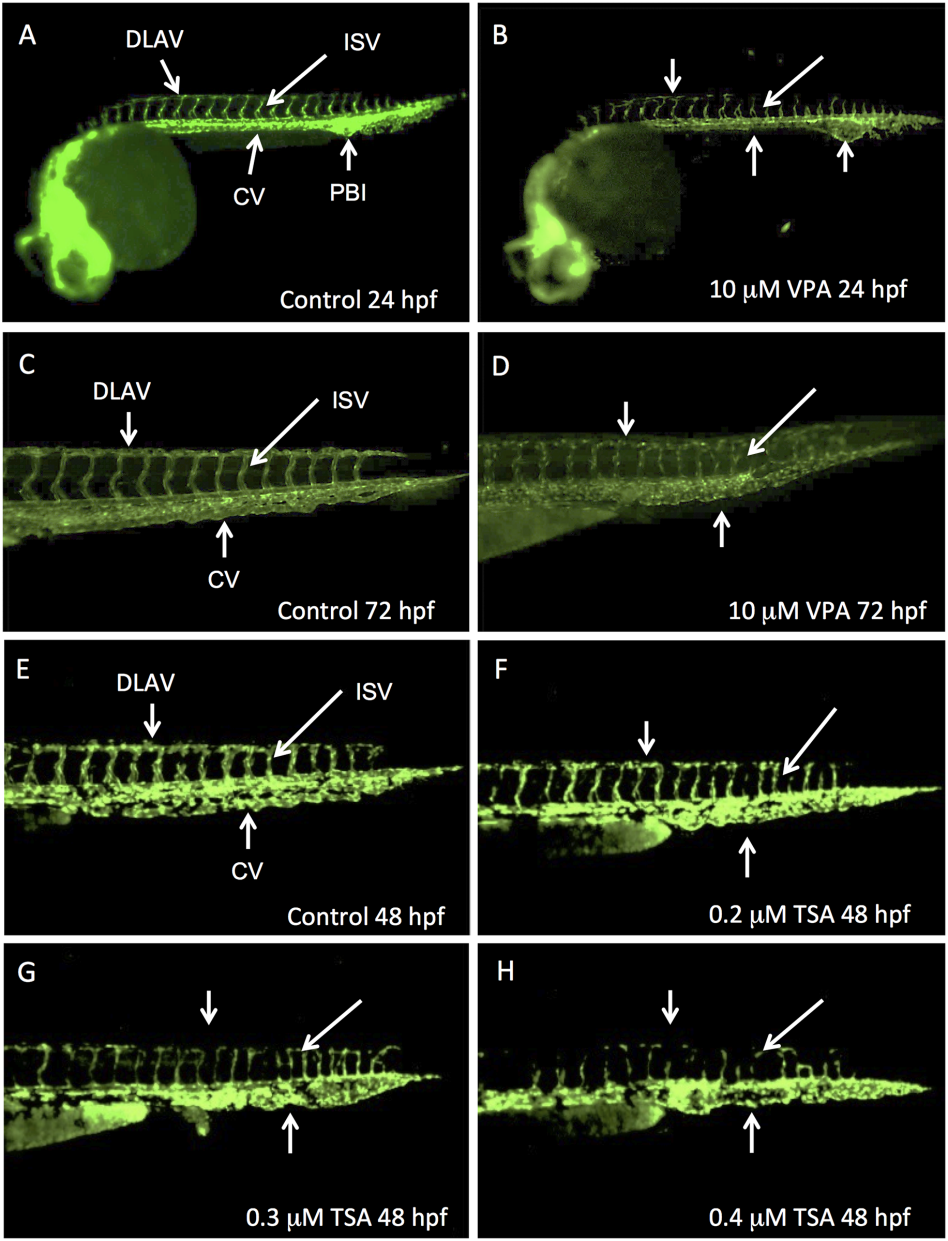
VPA and TSA perturb the development of vascular system. Lateral views of Fli1-EGFP whole embryos at 24 hpf (A, B) or of their caudal part at 48 hpf (E-H) or 72 hpf (C, D). Control embryos at 24 hpf (A), 48 hpf (E) and 72 hpf (C). Embryos treated with VPA 10 μM observed at 24 hpf (B) or 72 hpf (D). 48 hpf embryos treated with increased concentrations of TSA: 0.2 μM (F), 0.3 μM (G) or 0.4 μM (H). DLAV: dorsal longitudinal anastomotic vessel; ISV: intersegmental vessel; CV: caudal vein; PBI: posterior blood island.

For concentrations below 1 μM, we did not detect any effect of VPA exposure. At higher concentrations (starting at 2.5 μM), we observed dose-dependent vascular defects. At 24 hpf, the posterior blood island (also called caudal vein plexus) was larger than in control and the caudal vein was not clearly defined (Fig 1A and 1B). Furthermore, the dorsal longitudinal anastomotic vessel and the intersegmental vessels were malformed (Fig 1A and 1B). Later on, at 48 hpf, the vascular structure restored to normal for embryos treated with concentrations of VPA of 2.5 μM, 5 μM and 7.5 μM. These defects persisted at 72 hpf only when embryos were exposed at the highest concentration of 10 μM (Fig 1C and 1D).

For a concentration range of TSA between 1nM and 0.1 μM, there was no detected effect on treated embryos. By contrast, we observed a complete embryonic lethality from 1 μM TSA at 24 hpf, 0.75 μM TSA at 48 hpf and 0.4 μM TSA at 96 hpf. Therefore, we analyzed 48 hpf embryos that were exposed to “intermediate” concentrations between 0.1 μM and 1 μM. In these embryos, we observed abnormalities of vascular development (90% of embryos at 24hpf; 70% at 96hpf)). Compared to control (Fig 1E), the caudal vein was not remodeled to a clear vascular tube. In contrast with VPA, TSA did not affect the posterior blood island. We could also observe a delay in the formation of the dorsal longitudinal anastomotic vessel and the intersegmental vessels. These vessels were not well formed at 0.2–0.4 μM (Fig 1F, 1G and 1H) and absent at 0.5 μM. These effects were clearly dose-dependent: the higher concentration in TSA, the stronger the effect.

**Fig 2:**
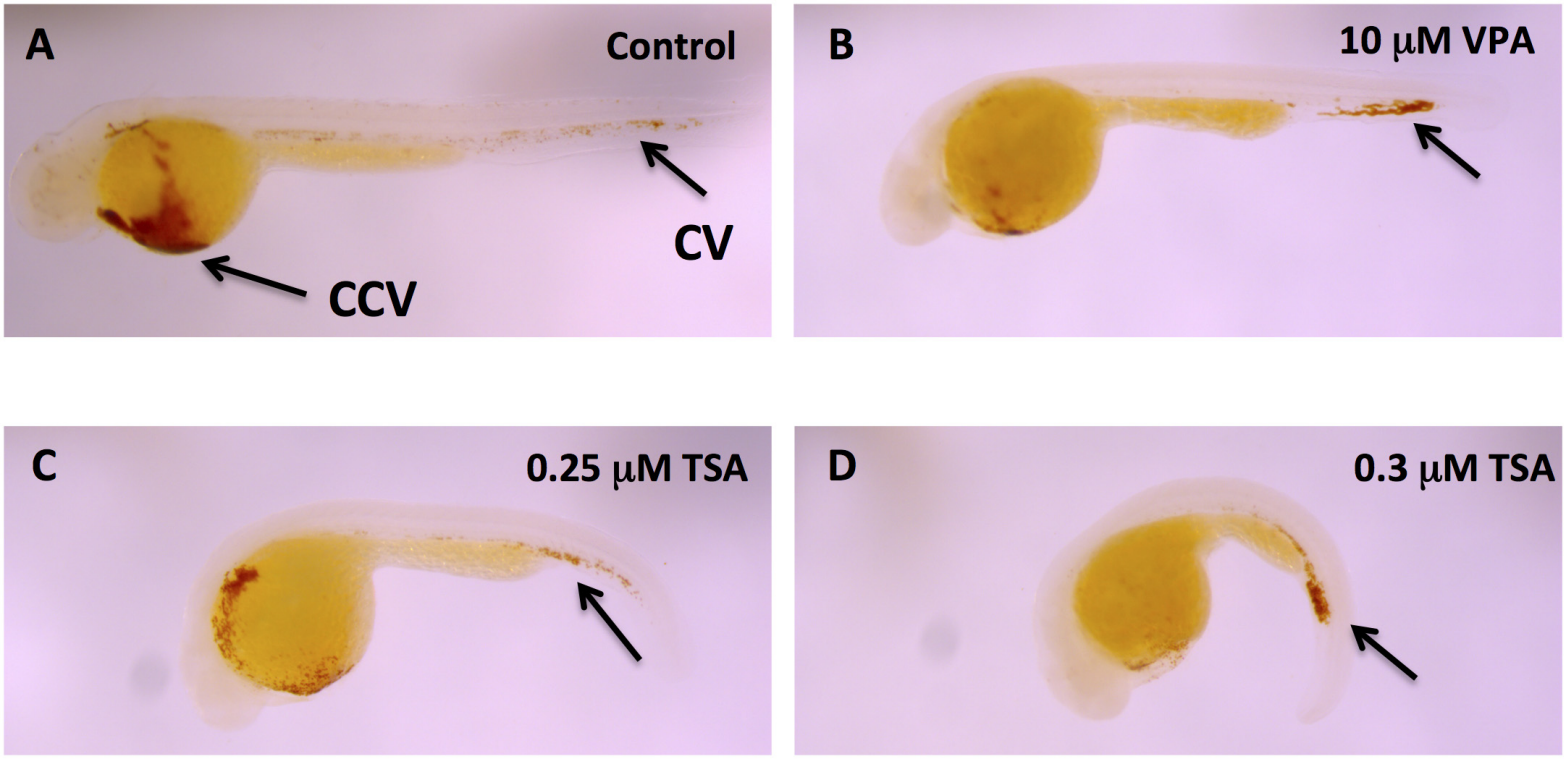
VPA and TSA impair hematopoietic cell migration. Erythrocytes revelation by hemoglobin localization using O-dianisidine staining in 48 hpf embryos. Control embryo (A). Embryos treated with 10 μM VPA (B), 0.25 μM TSA (C), or 0.3 μM TSA (D). CCV: common cardinal vein; CV: caudal vein.

These data show that both VPA and TSA perturb the vascular development in zebrafish, which is consistent with our qualitative visual observations of a slower circulation of blood cells and decreased heart beat rate in treated embryos. By 24 hpf, with the onset of heart contraction, primitive erythrocytes from the posterior hematopoietic tissue along the tail start to circulate over the yolk sac [33]. We therefore determined the localization of erythrocytes in 48 hpf embryos treated with TSA or VPA by revealing hemoglobin using O-dianisidine staining. We observed that these embryos have less hemoglobin in the yolk sac and an accumulation in the tail (Fig 2). In TSA treated embryos with concentrations greater than or equal to 0.3 μM, only the caudal vein was stained, and not the yolk (Fig 2D). This confirms the results obtained with the Fli1-EGFP transgenic line.

Our results therefore reveal that VPA and TSA disrupt the development of the vascular system in zebrafish embryos.

### VPA and TSA disrupt pancreatic development

**Fig 3:**
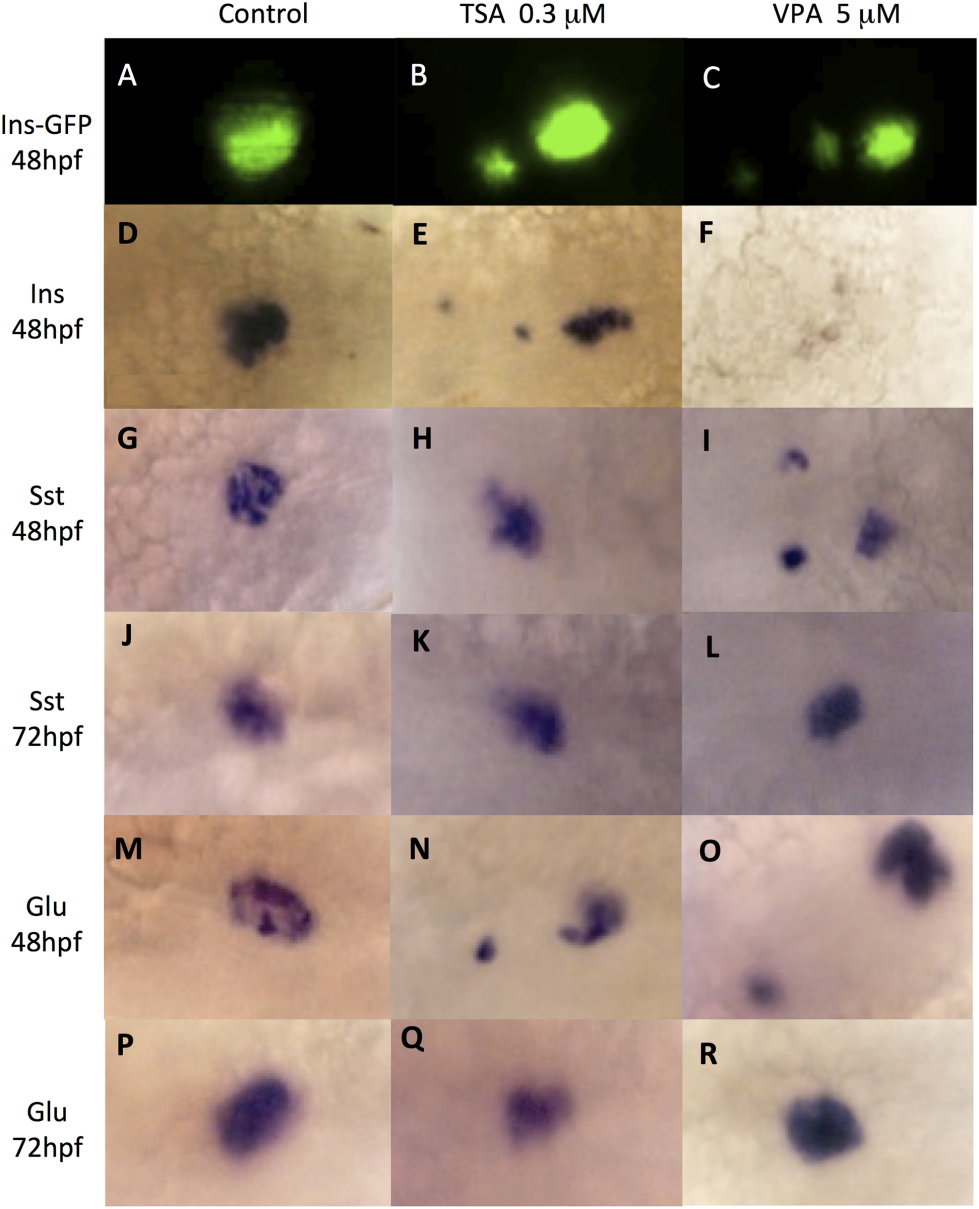
VPA and TSA disrupt cells clustering in endocrine pancreas. Cells of the endocrine pancreas are revealed in control embryos (A, D, G, J, M, P) or embryos treated with 0.3 μM TSA (B, E, H, K, N, Q) or 5 μM VPA (C, F, I, L, O, R). GFP fluorescence in β-cells of 48 hpf embryos of the insulin-GFP transgenic line (A, B, C). Whole-mount *in situ* hybridizations to detect the transcripts of: insulin in β-cells (D-F); somatostatin in δ-cells at 48hpf (G-I) or 72 hpf (J-L); glucagon in α-cells at 48hpf (M-O) or 72 hpf (P-R).

**Fig 4:**
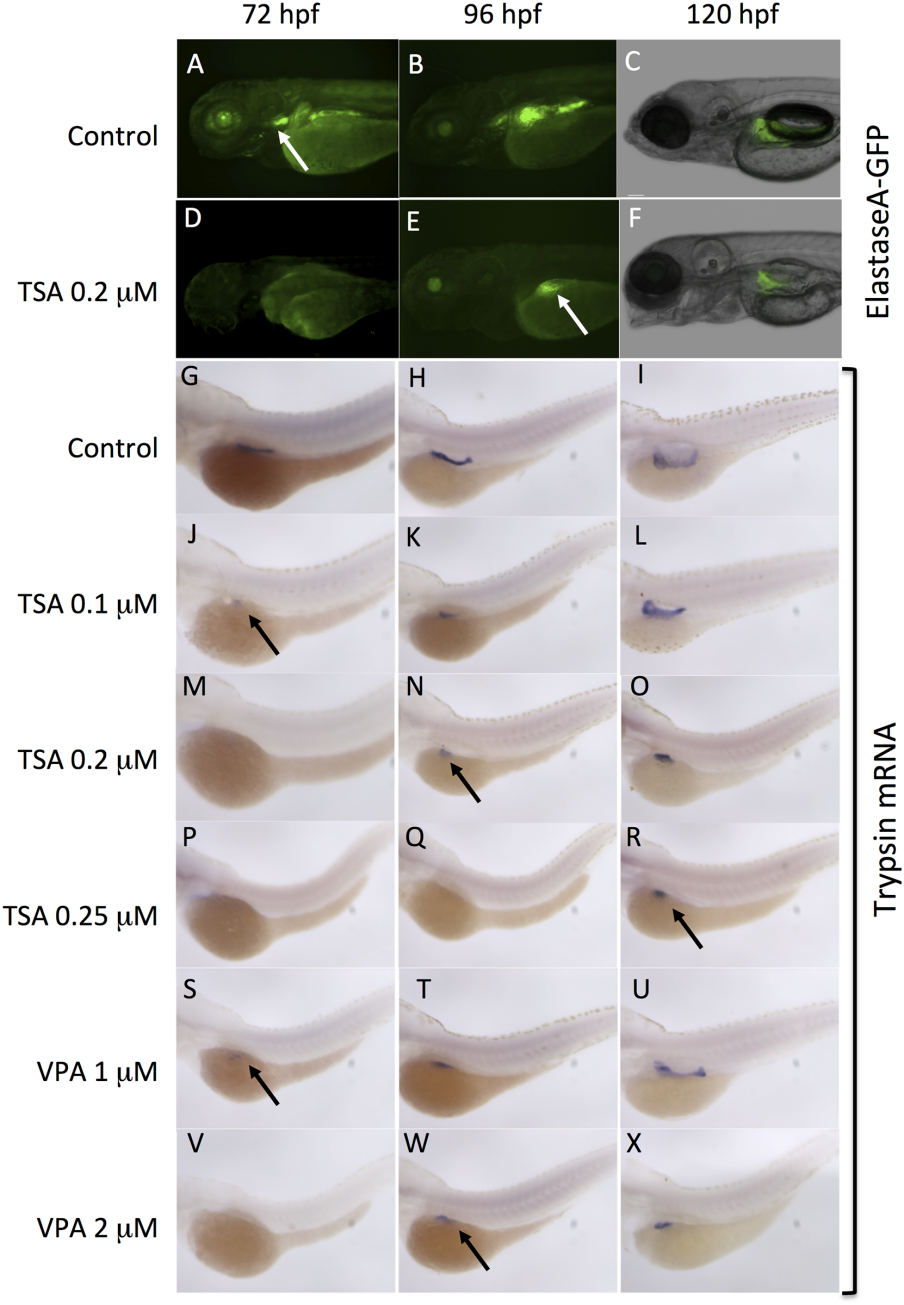
VPA and TSA delay the development of exocrine pancreas. The development of exocrine pancreas was analyzed at 72 hpf, 96 hpf and 120 hpf. GFP fluorescence in embryos of the ElastaseA-GFP transgenic line: controls (A-D), embryos treated with 0.2 μM TSA (D-F). White arrows indicate the exocrine pancreas. Whole-mount *in situ* hybridizations to detect trypsin transcripts (G-X) in differentiated pancreatic exocrine cells of control embryos (G-I), embryos treated with increasing concentrations of TSA (J-R) or VPA (S-X). Black arrows indicate the exocrine pancreas.

To understand the defects induced by VPA and TSA during pancreatic development, we used different markers expressed either in the endocrine pancreas (Fig 3), or in the exocrine pancreas (Fig 4). In zebrafish embryo, posterior endodermal precursor cells start to express the insulin gene by 15 hpf (12 somites) [34]. By 24 hpf, these cells cluster to form a dorsal bud that will give rise to the endocrine pancreas. The exocrine pancreas emerges by 32 hpf as a ventral bud. The dorsal and ventral buds fuse by 48 hpf [34,35].

We first analyzed the endocrine pancreas using the insulin-GFP transgenic line. In this line, GFP expression is under the control of a 4 kb promoter region of the insulin gene, which allows to follow the β-cells that express insulin [26]. The development of β-cells was observed from 24 hpf to 72 hpf in embryos treated at 5 hpf with VPA and TSA. At 48 hpf, whereas GFP expression is restricted to the dorsal bud in the control embryos (Fig 3A), a scattered expression in two or three spots was observed in embryos treated with TSA 0.3 μM (Fig 3B) and VPA 5 μM (Fig 3C). This effect was visible by 24 hpf (90% for VPA, 100% for TSA) and was still present at 96 hpf (77% for VPA, 90% for TSA). We observed the same disruption when treatments were applied at 10, 12 and 15 hpf, albeit at higher concentrations. Therefore, this abnormal clustering was not caused by a delayed development of the embryos. In order to confirm these observations, we performed whole-mount *in situ* hybridization to detect insulin transcripts. The results were consistent with the GFP expression for both TSA (Fig 3E) and VPA (Fig 3F). We therefore conclude that VPA and TSA can alter the clustering of insulin β-cells at low concentrations.

Three cell types contribute to the formation of the endocrine compartment of pancreas by the first day of zebrafish development: insulin α-producing β-cells, somatostatin-producing δ-cells and glucagon-producing α-cells [36,37]. As we have found that VPA and TSA have effects on the development of endocrine pancreas by monitoring β-cells presence, we also analyzed the expression of glucagon and somatostatin using whole-mount *in situ* hybridization. TSA had no effect on somatostatin-producing cells (Fig 3H and 3K). By contrast, in VPA treated embryos, somatostatin expression revealed that the clustering of δ-cells was not achieved at 48 hpf (Fig 3I), but only at 72 hpf (Fig 3L). A similar effect (no clustering before 72 hpf) was observed for glucagon-producing α-cells with both TSA (Fig 3N and 3Q) and VPA (Fig 3O and 3R).

These results show that VPA and TSA disrupt the clustering of endocrine cells during pancreas development.

We then examined whether these compounds can also affect the development of the exocrine compartment of the pancreas, which produces digestive enzymes that are secreted into the small intestine [27]. We first used an ElastaseA-GFP transgenic line to visualize the effect of TSA. In this line, GFP expression is controlled by a 1,875 bp promoter of the *elastaseA* gene (serine protease) that is an exocrine pancreas specific marker, from the third day (Fig 4A and 4G) throughout adulthood [38].

In embryos treated with TSA 0.2 μM, the GFP was not detectable at 72 hpf (Fig 4D). Later, at 96 hpf (Fig 4E) and 120 hpf (Fig 4F), the expression was visible but revealed that these embryos have a smaller exocrine pancreas, when compared to the control (Fig 4B and 4C). These results suggest that TSA delays the development of exocrine pancreas, resulting in a smaller size of the final organ. The same effect was observed with VPA (not shown).

In order to go further, we used *in situ* hybridization to detect the expression of trypsin gene in VPA and TSA treated embryos. Trypsin is a digestive enzyme that is expressed by 48 hpf in differentiated pancreatic exocrine cells [37]. Treatment with TSA 0.2 μM resulted in a delay on the expression of trypsin, which was not detected before 96 hpf (Fig 4M and 4N). At 120 hpf, the pattern revealed a small exocrine pancreas in the embryo (Fig 4O). These results perfectly match the data obtained with the elastaseA-GFP transgenic line (Fig 4D and 4F). In addition, we found that the effects of TSA were dose-dependent. Indeed, at lower concentration (0.10 μM), the onset of trypsin expression was visible by 72 hpf (Fig 4J) and the exocrine pancreas appeared normal at 120 hpf (Fig 4L). By contrast, embryos treated with higher concentrations of TSA (0.25 μM) lacked trypsin expression until 120 hpf and the exocrine pancreas remained small (Fig 4P and 4R). Similar dose-dependent effects were obtained with VPA, with a delay in the onset of trypsin expression and a reduction in the size of exocrine pancreas (Fig 4S and 4X).

In conclusion, the HDAC inhibitors VPA and TSA disrupt the development of endocrine pancreas and delay the formation of exocrine pancreas in zebrafish. The two compounds have specific dose-dependent effects.

### VPA and TSA induce liver defects and increased fat accumulation

Since VPA and TSA disrupt pancreatic development, we checked if low doses of these compounds could affect liver as well. Indeed, both organs develop from the posterior foregut and, later on, pancreatic hormones are important regulators of liver metabolism. Actually, it has already been shown that VPA and TSA impair liver development in zebrafish [39]. However, these non-teratogenic effects were obtained with unspecified concentrations (TSA) or concentrations higher than the one we used for pancreas (10–20 μM for VPA). We therefore decided to characterize the effects of TSA and VPA on liver at lower concentrations (<1 μM). To that end, we used the LFABP-EGFP transgenic line, in which the GFP is regulated by 2.8 kb promoter sequence of the gene encoding the liver fatty acid binding protein (LFABP). In this line, GFP expression is first visible at 48 hpf in the liver primordia and until adult stage [28]. In embryos treated with VPA and TSA at 0.2 μM, the liver was not detectable at 72 hpf and became visible at 96 hpf (S2 Fig). This defect is less severe than at higher concentrations, where no sign of GFP expression was detected until 120 hpf [39]. Therefore, both VPA and TSA can delay the development of liver at low concentrations.

**Fig 5:**
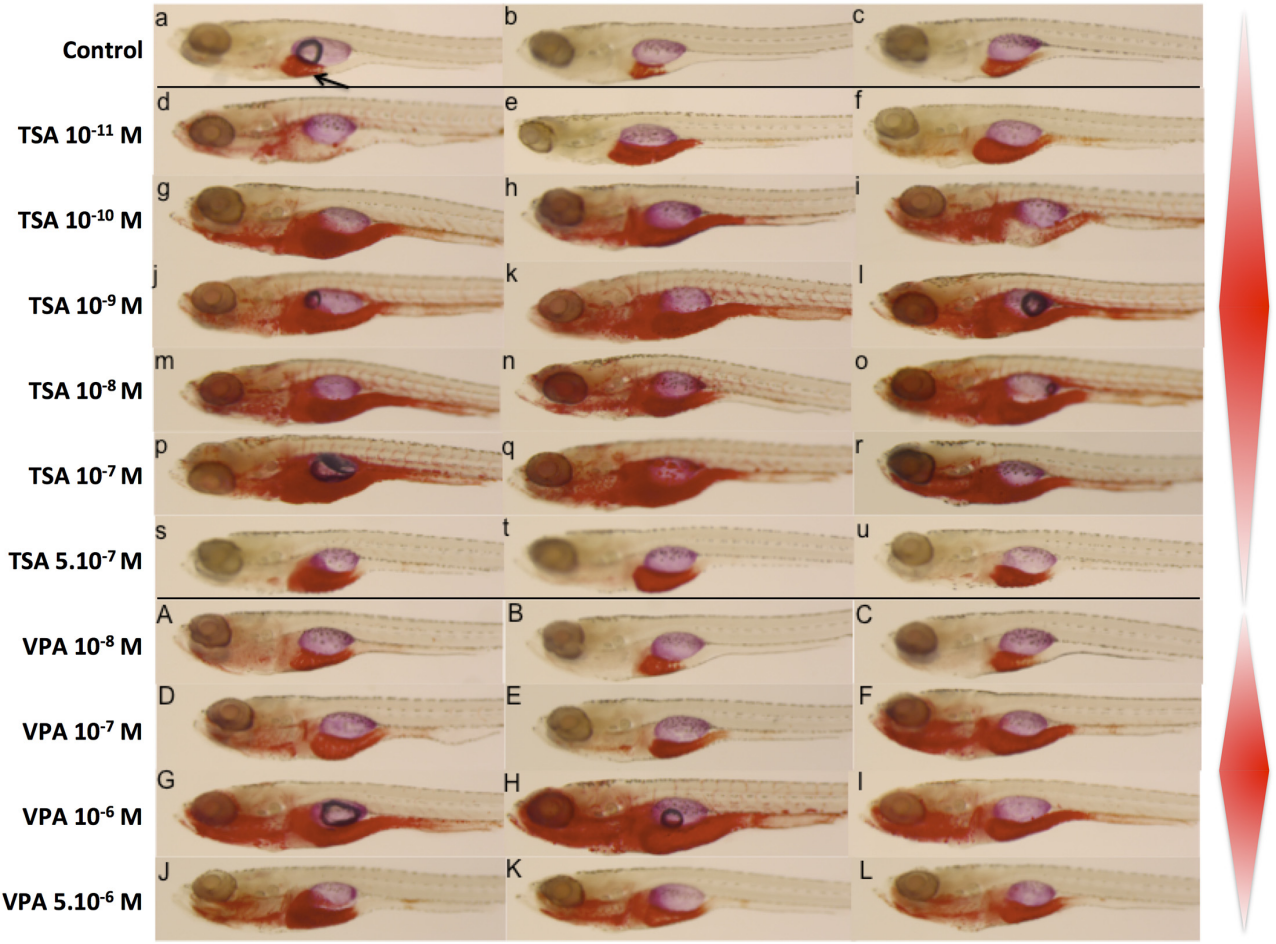
Lipid accumulation in larvae treated with VPA and TSA. Oil Red O staining shows the localization of neutral lipids in 10 dpf larvae that have been on a high fat diet between 6 dpf and 9 dpf. An arrow indicates the liver. Control embryos (a-c), embryos treated with increasing concentrations of TSA (d-u) or VPA (A-L). The red lozenges on the right schematize the lipid accumulation, highlighting the non-monotonic dose response. Note that the maximum accumulation is observed at intermediate concentrations.

Since pancreatic hormones are key regulators of liver metabolism [40], and because zebrafish liver is functional after hatching (48 hpf), we analyzed the effect of VPA and TSA on lipid accumulation in larvae submitted to a high fat diet (egg yolk) from 6 to 9 days post fertilization [24]. We used Oil red O staining that reveals neutral lipid accumulation. In TSA treated animals, more fat was accumulated in liver and gut region than in the control, even at the very low dose of 10^−11^ M (Fig 5d, 5e and 5f). At an intermediate concentration of 10^−9^ M TSA, additional strong staining was observed in jaw, anterior intestine, heart and blood vessels (Fig 5j, 5k and 5l). Strikingly, this effect was not visible at the highest concentration tested (5x10^-7^ M) (Fig 5s, 5t and 5u).

The effect of VPA on this end point was similar, although shifted to higher concentrations. Larvae treated at 10^−8^ M VPA were not different from the control (Fig 5A, 5B and 5C). By contrast, at 5x10^-6^ M VPA the larvae accumulated more fat in liver and gut region (Fig 5J, 5K and 5L). Once again, as for TSA, we observed additional staining in jaw, anterior intestine, heart and blood vessels at an intermediate concentration of 10^−6^ M VPA (Fig 5G, 5H and 5I).

Taken together, these results suggest that VPA and TSA effects on early development of endodermal organs translate into later effects at the physiological level, with a propensity to accumulate neutral lipids in a high fat diet situation. Surprisingly, our results also suggest that these effects may be an example of non-monotonic dose response.

### VPA and TSA inhibit pharyngeal teeth development

**Fig 6:**
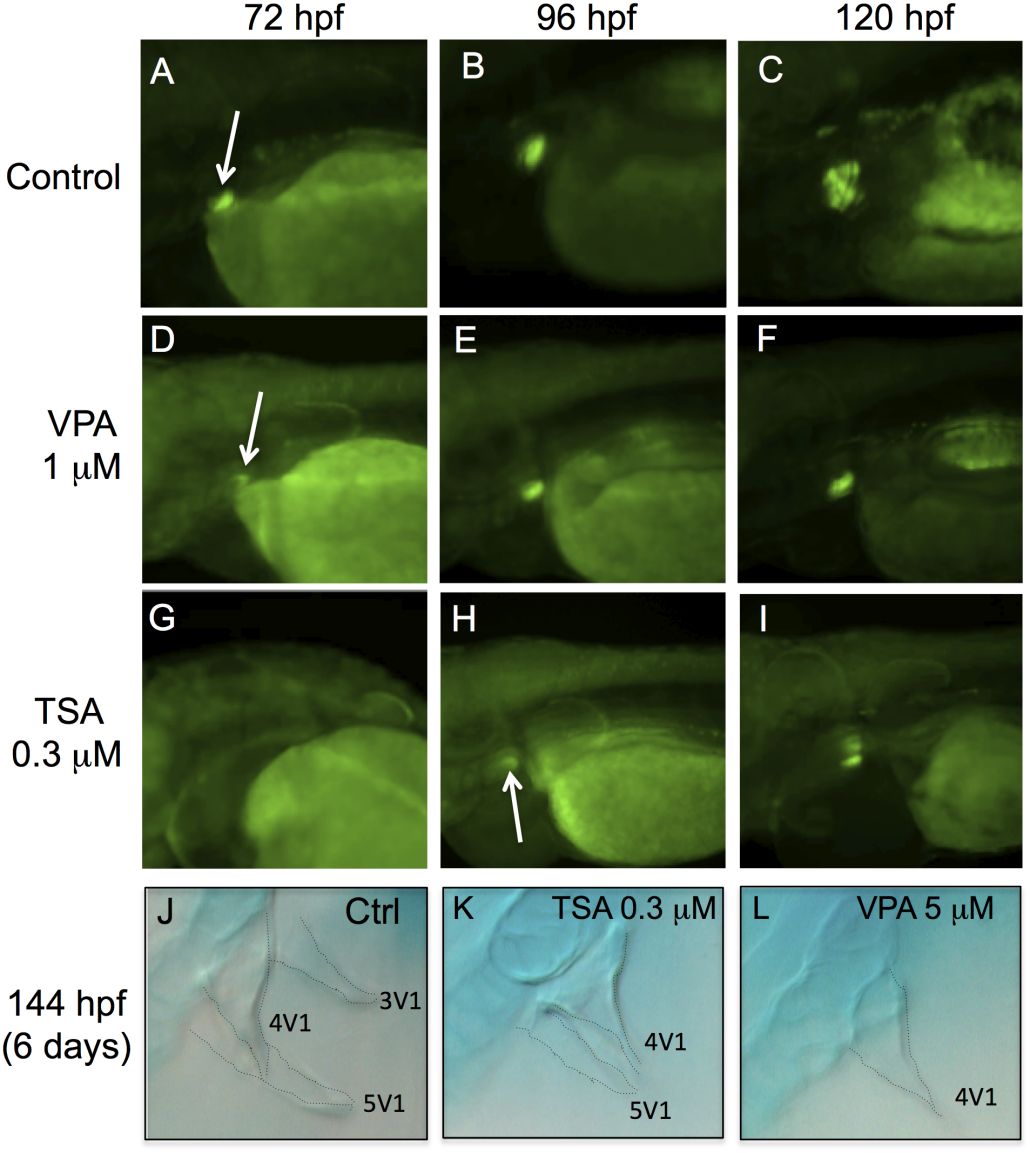
VPA and TSA delay the formation of pharyngeal teeth. The development of pharyngeal teeth was analyzed at 72 hpf, 96 hpf and 120 hpf. GFP fluorescence in embryos of the Dlx2b-EGFP transgenic line: controls (A-C), embryos treated with 1 μM VPA (D-F) or 0.3 μM TSA (G-I). White arrows indicate pharyngeal tooth germs. Alcian blue staining at 144 hpf in control embryos (J) or embryos treated with 0.3 μM of TSA (K) or 5 μM of VPA (L). Dotted lines highlight the contours of three teeth: 3V_1_, 4V_1_ and 5V_1_.

We analyzed the effects of VPA and TSA in the pharynx, by using the Dlx2b-EGFP transgenic line, which contains 4.1 kb of promoter of the distal-less-related transcription factor, Dlx2b [29]. In this line, the GFP fluorescence is detectable in the developing pharyngeal teeth of zebrafish by 60 hpf and is very strong by 72 hpf (Fig 6A).

At 72 hpf, the fluorescence was barely detectable in the pharyngeal tooth germ of VPA treated embryos (Fig 6D), and was absent in TSA embryos (Fig 6G). By 96 hpf, the expression of Dlx2b-EGFP was stronger in VPA embryos (Fig 6E), and became visible in TSA embryos (Fig 6H). However, a difference in staining at 96 hpf (4 days) suggested that the tooth germs were smaller than in the control (Fig 6B). This difference was still present at 120 hpf (5 days) (Fig 6F, 6G, 6H and 6I), suggesting that some teeth may be absent at this stage. We therefore performed alcian blue staining to count the teeth in 144 hpf (6 days) embryos. In controls, we observed the first tooth 4V_1_ of the first row as well as the two other teeth, 3V_1_ and 5V_1_ (Fig 6J). In TSA treated embryos we observed 4V_1_ and a small part of a second teeth 5V_1_ (Fig 6K). In VPA treated embryos, only tooth 4V_1_ was present (Fig 6L). Since TSA and VPA reduced the number of teeth without affecting the pharyngeal skeleton, this effect is probably not linked to a general developmental delay induced by the treatments.

These data show that both VPA and TSA can delay the formation of pharyngeal teeth in zebrafish.

## Discussion

Whereas the effects induced by endocrine disruptors (EDCs) on reproductive development are well known [41], their impact on other organs are comparatively understudied. Here, we report that one known EDC (TBBPA) and three pharmaceuticals drugs (diclofenac, TSA and VPA) effectively disrupt the normal development of zebrafish embryos.

### A screen for EDCs' developmental effects

Our strategy was designed to be rapid and straightforward. We used zebrafish because this complete multi-organ vertebrate system allows screening for early developmental effects, when embryos are particularly vulnerable to toxic and environmental influences [11]. Our screen was based on a static non-renewal test that minimizes embryo manipulation and insures that the exposure dose is not superior to the nominal concentration (the concentration given at the beginning of exposure). This was particularly important for us because we were interested by low-dose effects, namely well below the lethal dose. Therefore, our test was not designed to identify toxicity in the conventional sense, but rather to detect embryonic disruption. However, this strategy has obvious limitations: if a compound is rapidly degraded, its activity will not last for long and we may not detect effects that could be important in case of chronic exposure. Consequently, we have chosen compounds that are stable in water.

We also took advantage of the numerous zebrafish transgenic lines that allowed us to visualize the effects of small molecules on three distinct systems: the vascular, digestive and nervous systems. The estrogen-sensing reporter transgenic line cyp19a1b-GFP [42] or the ligand trap system PPARγ−LBD/DBD-GAL4 [24] would have been interesting for us only if their expression would have not been restricted to reproductive organs. Indeed, our goal was to focus on developmental process, and not on reproduction, which has been highly studied for a long time [41]. Ideally, this kind of study should include lines that are expressed in most of the physiological or developmental system of the developing fish. However, in addition to hamper the rapidity of the screen, such a comprehensive approach can also be limited by the availability of robust and healthy transgenic lines with high fertility. This is particularly a problem for maintaining homozygous transgenic lines that allow the easy quantification of GFP fluorescence [43]. All our lines were hemizygous, which implies that their progeny contained a mixture of embryos with different GFP signals. We therefore did not measure the fluorescence intensity. Overall, and despite the limits discussed above, we can safely conclude that our results confirm zebrafish as a powerful tool for rapid *in vivo* screening of small molecules and their effects on development.

In this screen, we tested nine molecules that represent different classes of substances, both in terms of use and mode of action. We selected six clear endocrine disruptors used as flame-retardants, herbicides, insecticides or plasticizer as well as a heavy metal. In addition, we selected three molecules used as antibiotic, anticonvulsants and anti-inflammatory drugs. These compounds cover the wide range of biological targets of EDCs and, hence, the complexity of their mode of action and effects. Five of the known EDCs (DEHP, chlordecone, atrazine, methoxychlor and cadmium) did not show any effects on zebrafish embryos, although their ability to disturb the reproduction of this species is well characterized [44–47]. It may not be surprising for DEHP and chlordecone since, to our knowledge, no study has ever reported an effect of these compounds on the development of zebrafish. By contrast, the results obtained with atrazine, methoxychlor and cadmium were unexpected, since these molecules can alter the development of zebrafish and other species (see discussion below). Plausible explanations for these differences include dosage of the compounds, timing of exposure and the suitability of transgenic lines and these possible differences are described below. In our rapid screen, we used low concentrations, started the treatments at 5 hpf and observed until 120 hpf. It is thus possible that effects on the development of zebrafish larvae would have been observed only with higher concentrations of these EDCs and/or after longer exposure. Alternatively, one might consider the penetrance of these compounds into zebrafish embryos and their partial decay during the exposure.

Atrazine is able to induce aromatase, which leads to an excess of estrogen synthesis. It can also activate the NR5A/SF1 nuclear receptor, possibly by regulating its phosphorylation [48,49]. At 50 μM, atrazine induced detoxification genes in mosquito larvae [50]. In zebrafish, a treatment with atrazine 20 μM just after fertilization could disturb the normal development (blood circulation and delays) [45]. By using the nkx2.2a-mEGFP transgenic line, a neurotoxic effect was also revealed when zebrafish embryos were treated at 10 μM atrazine at 3 hpf [46]. We did not detect an effect at the same concentration on the nervous system, possibly because our treatments started later at 5 hpf. Furthermore, we used different transgenic lines (α1-tubulin-EGFP, Shh-GFP, Brn3C-mGFP) that may not allow to visualize the type of effect observed with the nkx2.2a-mEGFP. Methoxychlor is an insecticide that behaves as an antagonist for human ERβ and an agonist for human ERα [51]. At high concentrations (300 μM), methoxychlor induces precocious hatching of salamander embryos [52] and retardation of cleavage and abnormal gastrulation of sea urchin [53]. In zebrafish embryos, a 24 hours treatment beginning at 72 hpf with methoxychlor 14 μM (near sublethal concentration) exhibited stress on the brain and spinal cord [44]. These authors used the huORFZ transgenic line, which is very effective to detect stress-related cellular processes induced by environmental toxicants. We did not detect any effect in our screen, probably because we used lower concentrations (10 μM maximum) and less specific lines. It is not surprising that we did not detect gastrulation defects as we treated only at 5 hpf, that is, after the onset of gastrulation. An opposite situation was seen with cadmium chloride (CdCl_2_), which had effects at 5 μM using the same huORFZ transgenic line, but not in our treatment at 10 μM. It seems therefore that the *huORFZ*-GFP transgenic line used by these authors is a more sensitive tool to detect cadmium stress on the development of olfactory epithelium, skin, lateral line and pronephric duct [44].

In conclusion, a positive and simple explanation for the lack of effects of known EDCs in our screen could be that low-doses of these compounds do not have the ability to disrupt embryogenesis of zebrafish. If confirmed, these results would nevertheless be relevant to the field of toxicology, because the lack of deleterious effects of a given molecule is useful information for risk assessment.

The vascular system (Fli1-EGFP reporter line) was affected by four molecules: TBBPA, diclofenac, VPA and TSA. However, these compounds do have not the same effects, suggesting that they act through different targets. Tetrabromobisphenol A (TBBPA) is used primarily as a flame retardant and was shown to be an anti-androgenic molecule [23]. It also acts as an obesogene through its agonist activity on PPARγ [24]. Zebrafish embryos treated with 0.75 μM TBBPA at 3 hpf had smaller body length, curved tails and malformed trunk [54]. We observed the same problems, but at higher concentration (2.5 μM) and later exposure time (5 hpf). Furthermore, using Fli1-EGFP, we detected a new effect of TBBPA on the vascular development of zebrafish embryos (S1 Fig). Since DEHP, which also targets PPARγ, had no effect, it is possible that TBBPA action on vasculature is not mediated by its activation of PPARγ, but by another target. Alternatively the difference of effect can be linked to a difference in the metabolism and stability of the two compounds in the embryo. Diclofenac is an inflammatory drug that blocks the synthesis of prostaglandins by inhibiting cyclooxygenases [55]. This drug induces teratogenic effects on rat embryos [56]. Diclofenac treated zebrafish embryos have defects on their central nervous system, yolk sac, heart and during hatching [57,58]. It has been shown that inhibition of COX-1 signaling by other drugs results in defective vascular tube formation and shortened intersomitic vessels in the posterior body region [59]. Our results reveal a broad effect of diclofenac on the whole cardiovascular development in zebrafish (S1 Fig).

### VPA and TSA induce a wide array of developmental defects

The pharmaceutical drugs VPA and TSA elicit complex effects at multiple levels during development. Both molecules are inhibitors of histone deacetylases (HDACs), which are recruited by transcription factors (especially nuclear receptors) and associated with transcriptional repression [60,61]. While VPA selectively inhibits HDACs I, TSA is an inhibitor of HDACs I and II. Although valproic acid has rarely been found in water surface, its presence in effluent samples has suggested that it should be taken into consideration in wastewater analyses [62,63]. The high consumption and limited metabolism of both VPA and TSA make them potentially high risk compounds to the aquatic environment [62]. We observed that zebrafish embryos are more sensitive to TSA than VPA. This result is consistent with studies on *Xenopus*, another screening model. Indeed, 200 μM VPA and 0.1 μM TSA had strong teratogenic effects (shorten axis, crooked tail, heart) on *Xenopus* embryos treated from stage 21–22 until stage 32–33 for 18h, which correlates with their ability to inhibit HDACs [64]. It has been shown that TSA (unspecified dose) and VPA (20 μM) affect angiogenesis, liver and exocrine pancreas formation of zebrafish, but not endocrine pancreas [39]. In our study, lower concentrations of VPA (5–10 μM) and TSA (0.25–0.5 μM) showed effects on angiogenesis, liver and exocrine pancreas. They may also impair intra-embryonic hematopoietic cell migration. In addition, these treatments also affected pharyngeal teeth and endocrine pancreas. The discrepancy regarding endocrine pancreas is surprising and raises the intriguing possibility of a specific low-dose effect of TSA and VPA. This may be clinically relevant, since inhibition of HDACs is a promising therapy against insulin-resistant diabetes [65]. The effect on teeth is more easily explained by the fact that HDAC-1 is required for the normal formation of craniofacial cartilage of zebrafish [66]. Our team has shown that retinoic acid receptors (RARs) are playing an important role in pharyngeal teeth development [15]. Since HDAC can bind to corepressors of nuclear receptors [67,68], it is possible that the effects of VPA and TSA on pharyngeal teeth development are due to the HDAC-mediated disruption of the retinoid signaling pathway [69].

Apart from the embryonic problems, we discovered that TSA and VPA also induced fat accumulation in 10 dpf zebrafish larvae. It seems therefore that the early developmental defects induced by these drugs could result in functional abnormalities later in life. In most cases, HDAC are recruited at specific promoters by transcription factors, especially nuclear receptors. It is known that inhibition of HDAC activity activates PPARγ [68]. So it would be interesting to study whether the TSA and VPA fat accumulation effects are mediated by an HDAC-mediated disruption of PPARγ during adipogenesis.

## Conclusion

In conclusion, we have shown that zebrafish transgenic lines are powerful models to perform rapid *in vivo* screens of the developmental alterations caused by small chemicals present in the environment. These exogenous substances may cause adverse health effects on intact adult organisms. In this article, we have provided evidences that both TSA and VPA alter organogenesis during specific developmental windows, at low concentrations and maybe sometimes with non-monotonic dose response. As such, they would share important features with endocrine disruptors, and may thus be called “embryonic disruptors”.

## Materials and Methods

### Zebrafish stocks

Breeding fish of the AB-TU wild-type strain as well as transgenic lines were reared at 28–29°C under a 8-h dark/16-h light cycle and staged as described [70]. Healthy adult female and male fish were mated at ambient temperature in a tank the night before spawning. Embryos were collected after spawning. Unfertilized eggs were manually separated from fertilized eggs. 1-phenyl-2-thiourea (PTU) at a final concentration of 0.2 mM was added to the embryos to prevent the development of endogenous pigments. The Institutional Animal Care and Use Committee at the Université de Lyon approved the protocols used for the experiments. All efforts were made to minimize their suffering. The transgenic lines used in this study are described in Table 1.

### Chemical exposure of embryos

Valproic acid (VPA, CAS No: 1069-66-5) was purchased from STEMGENT. All the other chemicals were purchased from Sigma-Aldrich: atrazine (CAS No: 1912-24-9), tetrabromobisphenol A (TBBPA, CAS No: 79-94-7), methoxychlor (CAS No: 72-43-5), Bis(2-ethylhexyl) phthalate (DEHP, CAS No: 117-81-7), cadmium chloride (CdCl_2_, CAS No: 10108-64-2), diclofenac (CAS No: 15307-79-6), chlordecone (CAS No: 143-50-0) and trichostatin A (TSA, CAS No: 58880-19-6). Stock solutions 10^−2^ M were prepared in dimethyl sulfoxide (DMSO), ethanol (ETOH) or distilled water and stocked at -20°C. Fresh dilution of chemicals were prepared before the experiments.

The crosses of transgenic lines (Table 1) were made at a ratio of 1 male to 1 female. Adults were hemyzigous. The spawns were treated further only if at least 75% of the embryos were fluorescent (either homozygous or hemizygous transgenic embryos). Thirty embryos 5 hpf (hours post-fertilization) were placed in each well of a 6-well plate, containing 5 ml of various concentrations (10^−3^ μM to 10 μM) of a chemical diluted in medium solution E3 for embryos (NaCl 5 mM; KCl 0.17 mM; CaCl_2_ 0.33mM; MgSO_4_ 0.33 mM; methylene blue 3%; pH 7,2). Control embryos were exposed to E3 (CdCl_2_) or solvents (DMSO: TSA, VPA, TBBPA, DEHP, atrazine, methoxychlor; ethanol: diclofenac, chlordecone) not exceeding 0.01% in the medium. Controls were made with the solvent only at a dose identical to the highest used for the treatments. Embryos were incubated at 28°C for 1 to 5 days (depending on the transgenic lines: see Table 1). Solutions were not changed during the overall experiment (static non-renewal test) and dead embryos were removed. The chorion was removed manually at 24 hpf to allow visualization of fluorescence. Each experiment was repeated at least three times. GFP expression was observed in live embryos under fluorescence macroscope (Leica Z16 APA A) equipped with a digital camera (Photometrics CCD CoolSNAP ES monochrome). Images were processed using Adobe Photoshop.

### Whole-embryo staining for globin expression

O-dianisidine staining with embryos of zebrafish at 48 hpf was performed as previously described [71].

### Whole-mount *in situ* hybridization

Whole-mount *in situ* hybridization of zebrafish embryos were performed as previously described [72]. Digoxigenin riboprobes of insulin, somatostatin, glucagon and trypsin (cDNA clones provided by Y. Gibert) were synthesized using DIG RNA labeling kit (Roche). Embryos were mounted in 3% methylcellulose medium and observed with an AXIOIMAGER microscope (Zeiss).

### Fat accumulation and lipid staining

Prior to Oil red O staining, embryos were submitted to a high fat diet composed of cooked egg yolk. Twenty wild type embryos at 5 hpf were placed in each well of a 6-well plate, containing 5 ml of medium solution E3. At 3 days post-fertilization, larvae were treated with different concentration of TSA and VPA. From 6 to 9 days post-fertilization, the larvae were fed each morning with 30 ml of high fat diet (2.4% egg yolk). At 10 days, larvae were fixed and neutral lipid accumulation was revealed with 0.3% Oil red O staining [24], during 4 hours. After washing, larvae were stored in 70% glycerol.

### Embryo staining for cartilage

Alcian blue staining was performed essentially as described by Walker and Kimmel [73], with minor modifications. 6-days fixed embryos were incubated with 0.1% alcian blue solution for 2 hours, then incubated twice for 30 minutes in a solution of 70% ethanol, 0.37% HCL. After clearing in 10% trypsin solution for 1 hour, embryos were transferred to 50% KOH for 1 hour and 0.25% KOH, 50% glycerol for 1 hour. Embryos were stored in 90% glycerol.

## Supporting Information

S1 Fig Developmental effects of diclofenac and TBBPA. Fli1a-EGFP. Untreated 48 hpf embryo (A) and embryo treated with 10 μM diclofenac (B) or 2.5 μM of TBBPA (C).
(PPTX)

S2 Fig VPA and TSA delay liver development. The development of liver was analyzed at 72 hpf, 96 hpf and 120 hpf. GFP fluorescence in embryos of the LFABP-EGFP transgenic line: controls (A-C), embryos treated with 0.2 μM TSA (D-F) or 0.2 μM VPA (G-I). White arrows indicate liver primordia.
(PPTX)

## Abbreviations

EDC: endocrine disrupting chemical
GFP: green fluorescent protein
TBBPA: tetrabromobisphenol-A
TSA: trichostatin A
VPA: valproic acid
HDAC: histone deacetylase
hpf: hours post-fertilization
dpf: days post-fertilization
