# Supplementary figures and images for: In Vivo Screening Using Transgenic Zebrafish Embryos Reveals New Effects of HDAC Inhibitors Trichostatin A and Valproic Acid on Organogenesis
Source: PLoS One. 2016 Feb 22;11(2):e0149497. doi: 10.1371/journal.pone.0149497 (PMC4763017; doi:10.1371/journal.pone.0149497)

## Slide 1
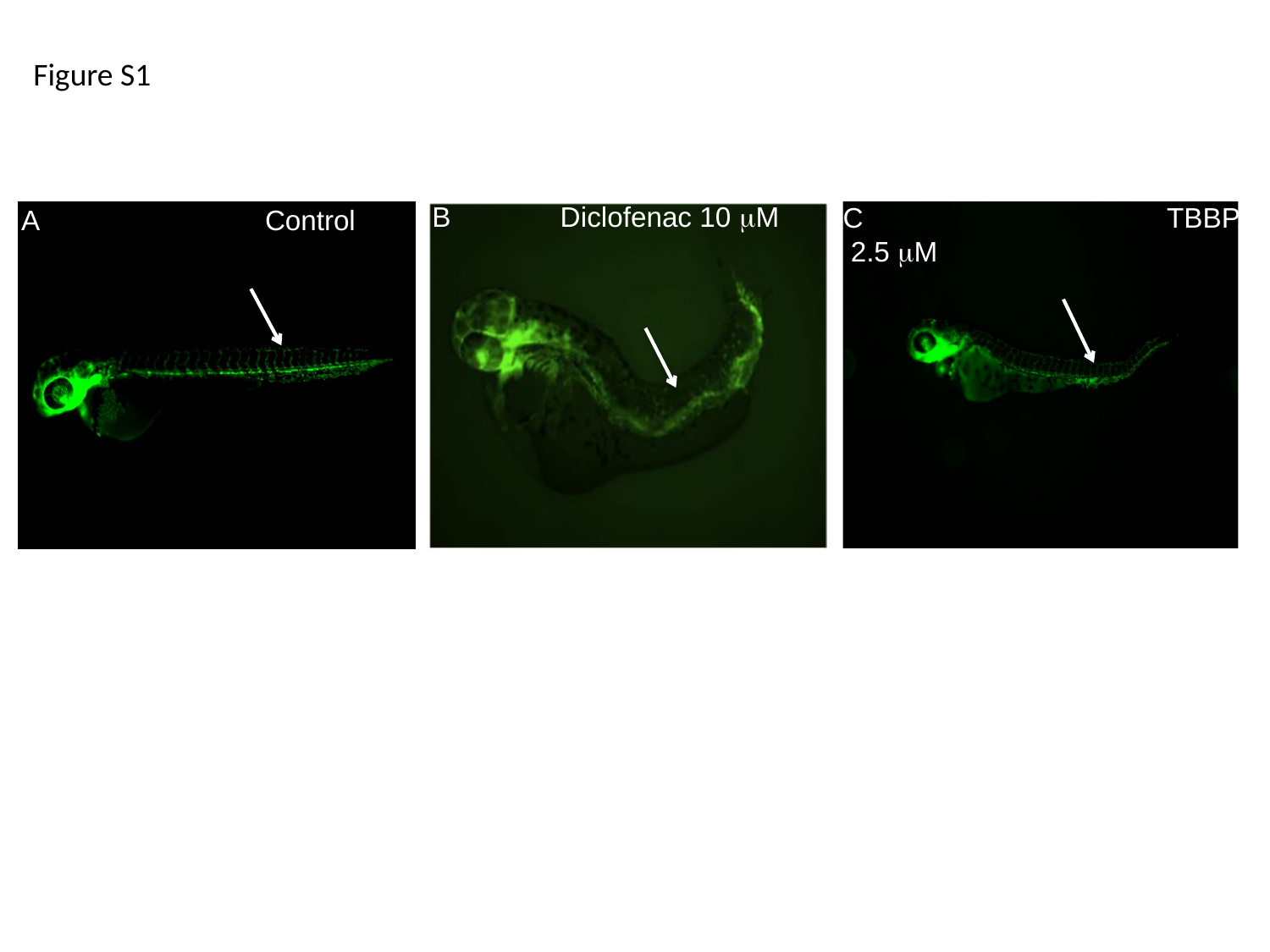

Figure S1
B Diclofenac 10 mM
C 		 TBBPA 2.5 mM
A Control

Supplement: S1 Fig — Fli1a-EGFP. Untreated 48 hpf embryo (A) and embryo treated with 10 μM diclofenac (B) or 2.5 μM of TBBPA (C). (PPTX) [file pone.0149497.s001.pptx]

## Slide 1
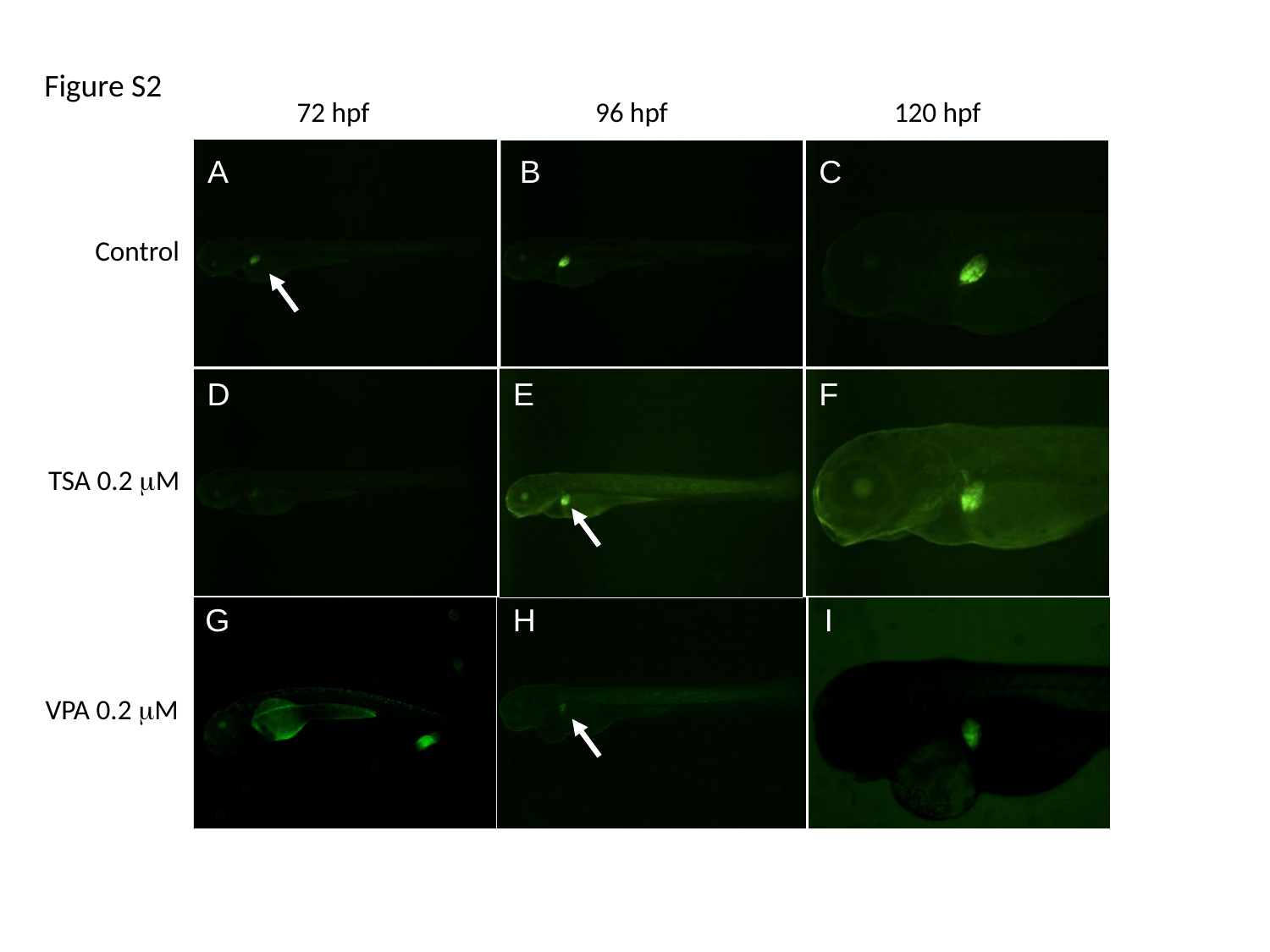

Figure S2
72 hpf
96 hpf
120 hpf
A
B
C
Control
D
E
F
TSA 0.2 mM
G
H
I
VPA 0.2 mM

Supplement: S2 Fig — The development of liver was analyzed at 72 hpf, 96 hpf and 120 hpf. GFP fluorescence in embryos of the LFABP-EGFP transgenic line: controls (A-C), embryos treated with 0.2 μM TSA (D-F) or 0.2 μM VPA (G-I). White arrows indicate liver primordia. (PPTX) [file pone.0149497.s002.pptx]
